# Earning pocket money and girls’ menstrual hygiene management in Ethiopia: a systematic review and meta-analysis

**DOI:** 10.1186/s12905-022-01855-2

**Published:** 2022-07-04

**Authors:** Biniyam Sahiledengle, Daniel Atlaw, Abera Kumie, Girma Beressa, Yohannes Tekalegn, Demisu Zenbaba, Demelash Woldeyohannes, Fikreab Desta, Tesfaye Assefa, Daniel Bogale, Fikadu Nugusu, Kingsley Emwinyore Agho

**Affiliations:** 1 Department of Public Health, School of Health Sciences, Madda Walabu University Goba Referral Hospital, Bale-Goba, Ethiopia; 2 Department of Human Anatomy, School of Medicine, Madda Walabu University Goba Referral Hospital, Madda Walabu University, Bale-Goba, Ethiopia; 3 Department of Community Health, College of Health Science, Addis Ababa University, Addis Ababa, Ethiopia; 4 Department of Public Health, College of Medicine and Health Science, Wachemo University, Hossana, Ethiopia; 5 Department of Nursing, School of Health Science, Madda Walabu University Goba Referral Hospital, Bale-Goba, Ethiopia; 6 College of Health Sciences, Arsi University, Asela, Ethiopia; 7 School of Health Sciences, Western Sydney University, Penrith, NSW 2571 Australia; 8 Translational Health Research Institute (THRI), Campbelltown Campus, Western Sydney University, Penrith, NSW 2571 Australia

**Keywords:** Menstrual hygiene, Adolescent girls, Pocket money, Sanitary pad, Ethiopia

## Abstract

**Background:**

Many adolescent girls in Ethiopia and elsewhere missed school during their monthly cycles due to a lack of affordable menstrual absorbent materials or money to buy sanitary pads. So far, few studies have looked into the relationship between earning pocket money and maintaining good menstrual hygiene. Hence, this systematic review and meta-analysis aimed to synthesize the best available evidence regarding the association between earning pocket money and menstrual hygiene management among adolescents in Ethiopia.

**Methods:**

We systematically searched PubMed, Hinari, Science Direct, Cochrane Library, ProQuest, POPLINE, African Journal Online, Direct of Open Access Journals, and Google Scholar for studies examining the association between earning pocket money and menstrual hygiene management among adolescent girls in Ethiopia, without restriction in a publication year. The Joanna Briggs Institute quality assessment tool for the cross-sectional studies was used to assess the quality of included studies. A prefabricated checklist, including variables: first author, publication year, sample size, type of questionnaire, and the region was used to extract data from the selected articles. A random-effect meta-analysis model was used to estimate the pooled odds ratio (OR) of the association between earning pocket money and menstrual hygiene management. The heterogeneity and publication bias was assessed by using I^2^ test statistics and Egger’s test, respectively.

**Results:**

Data from nine studies involving 4783 adolescent girls were extracted. The meta-analysis revealed that adolescent girls who earned pocket money from their parents or relative had 1.64 times higher odds of having good menstrual hygiene management than their counterparts [pooled OR = 1.64, 95% CI: 1.16–2.34, I^2^:66.7%, n = 7 (number of studies)]. Similarly, the likelihood of having good menstrual hygiene management was lower by 49% among adolescent girls who did not receive any pocket money from their parents compared to their counterparts (pooled OR = 0.51, 95% CI: 0.35–0.74, I^2^:48.4%, n = 2).

**Conclusions:**

The findings revealed that adolescent girls who earned pocket money were more likely to practice good menstrual hygiene management. Progress toward better menstrual hygiene will necessitate consideration of this factor.

**Supplementary Information:**

The online version contains supplementary material available at 10.1186/s12905-022-01855-2.

## Background

Menstruation is a normal part of a woman's sexual health during her childbearing years; however, girls are almost globally stigmatized and discriminated against for this natural process [1]. Menstrual hygiene management (MHM) refers to the use of clean menstrual management material to absorb or collect menstrual blood by women and adolescent girls. It is a part of overall sexual and reproductive health rights, based on all individuals' ability to decide over their bodies and live healthy and productive lives [2–4].

Lack of adequate water, sanitation, and hygiene (WASH) facilities, as well as limited access to affordable menstrual absorbent materials, cause adolescent girls' menstrual hygiene needs to go unmet in low-income settings [5]. In many cases, adolescent girls in low-income settings have developed their own menstrual hygiene practices to cope with menstruation [6]. These management techniques include the use of homemade clothes, old rags, underwear, sponges, and other poor materials for their periods [7–10]. These management techniques can be unsanitary and risky [6]. Poor menstrual hygiene can lead to a range of reproductive tract infections, such as urogenital infections [6, 11]. Torondel et al. suggested that lower reproductive tract infections were protected by changing absorbent pads more frequently [11]. According to UNICEF, girls should also change their menstrual absorbent materials three or four times a day [12].

The cost of commercial sanitary pads, a lack of water and latrine facilities, and poor knowledge of menstruation are important impediments to schoolgirls managing their periods [13–16]. Furthermore, girls in SSA miss school during menstruation due to a lack of menstrual products, physical pain, and fear of smelling or leaking blood on their school uniform [7, 17–19]. Sadly, in certain situations, girls engage in transactional sex to purchase sanitary pads and obtain pads from boyfriends to manage their menses [18–21]. According to a Kenyan qualitative study, girls' monthly struggle to buy sanitary pads can lead to sexual risk behaviors and raise the risk of STIs [21]. A cluster randomized controlled trial (RCT) in rural Kenya reported that providing menstrual hygiene management materials was associated with a lower risk of sexually transmitted infection (STI) [22].

For many adolescent girls in Ethiopia, the onset of menstruation could result in some amount of embarrassment and even fear due to poor family support [1, 23]. This is exacerbated further by society's negative attitudes, societal norms, and limited access to menstruation absorbent materials [1, 7, 10, 13]. Menstruation causes 32% of Ethiopian girls to miss one or more days of school each month [2]. Moreover, 48.9% of Ethiopian adolescent girls practiced poor menstruation hygiene [24]. There has been increasing evidence on the association between earning pocket money and good menstrual hygiene management [10, 23, 25–31]. However, existing studies have been small-scale or localized by area, and the conclusions made by those studies are inconsistent. For this reason, unequivocal evidence was not drawn at the national level. Therefore, we aimed to synthesize the best available evidence, and quantify the strength and direction of the association between earning pocket money and menstrual hygiene management among adolescents in Ethiopia. Evidence on the pooled estimate has the potential to move menstrual management forward.

## Methods

The Preferred Reporting Items for Systematic Reviews and Meta-Analysis (PRISMA) [32] were used to prepare and present this systematic review and meta-analysis (Additional file 1).

We employed the PEO (Population, Exposure of interest, Outcome) technique to establish inclusion and exclusion criteria.

### Eligibility criteria

- *Population* Adolescent girls (age 10–19).
- *Exposure of interest* Earning pocket money.
- *Outcome of interest* Good menstrual hygiene management.
- *Study designs* All observational study designs reporting the association between earning pocket money and menstrual hygiene were considered. In addition, studies that presented an adjusted odds ratio (OR) to assess the association between earning pocket money and menstrual hygiene management were considered for inclusion in the meta-analysis.
- *Study setting* Only studies conducted in Ethiopia.
- *Publication status* Both published and unpublished studies were considered.
- *Study period* There was no restriction on the publication date.
- *Language* Articles published in the English language were considered.
- *Year of publication* All publications reported up to September 15, 2021, were considered.

### Exclusion criteria

Study design: systematic reviews, commentaries, letters to editors, short communications, and qualitative studies were excluded. We also excluded articles, which were not fully accessed.

### Information sources

The following databases were subjected to a thorough search with no time limit. PubMed/MEDLINE, Science Direct, Google Scholar, Hinari, ProQuest, Directory of Open Access Journals, POPLINE, and Cochrane Library from inception to September 15, 2021.

### Searching strategy

The following key search terms and Medical Subject Headings [MeSH] were used *("Adolescent [MeSH Terms], OR Adolescents [Text Word], OR adolescence, OR puberty, OR peer, OR school”) AND (“Menstruation [MesH], OR menstrual, OR menses” AND “Hygiene [MesH], OR hygiene, OR hygienically, OR sanitation, OR sanitary, OR Feminine ‘’Hygiene Products’’ [MesH], OR ‘’Menstrual Hygiene Products’’ [MesH]”) AND (“Ethiopia”)* separately or in combination with the Boolean operator’s terms “AND” and “OR” (Additional file 2). The electronic database search was supplemented with gray literature searches via Google scholar and Google searching. Some research centers, including the Addis Ababa Digital Library, were searched to find gray literature in the field of our systematic review and meta-analysis. A secondary search method is known as "footnote chasing" has been used to identify relevant articles.

This systematic review and meta-analysis include all articles published till September 15, 2021, and the search was conducted between August 15 and September 15, 2021.

### Study selection process

Two investigators (BS and DA) independently screened and identified eligible articles by title, abstract, and full text against preset inclusion and exclusion criteria. The two authors (BS and DA) compiled the screened articles, and disagreements between them were resolved by discussion. In this review, all the searched articles were exported into the EndNote version X8 software, and subsequently, the duplicate articles were removed. Screening of retrieved article titles, abstracts, and full-text quality was conducted independently by two review authors (BS and DA) based on the eligibility criteria.

### Data collection process

Microsoft Excel was used to extract the data. Two authors (BS and DA) retrieved all necessary data independently using a predefined data extraction form. Any disagreements that arose during the data abstraction process were settled through discussion. The primary author, publication year, study design, study area, sample size, response rate, the proportion of good menstrual hygiene management, and confounder adjusted OR were all included in the data extraction format. EndNote version 8 reference manager software was utilized to collect search outcomes and remove duplicate articles.

### Risk of bias assessment of the studies

The Joanna Briggs Institute (JBI) quality evaluation method was used to assess the quality of the included studies [33]. The quality of the included studies was assessed independently by two reviewers (BS and DA). The evaluation tool has nine parameters. Failure to satisfy any of the parameters resulted in a 1, otherwise in a 0. We agreed to assign a 1 to an item when the information provided was insufficient to make a decision (a failure to satisfy a specific item or unclear). Bias risks were categorized as low (total score of 0 to 2), moderate (total score of 3 or 4), or high (total score of 5 or higher) [33].

### Synthesis of results

For statistical analysis, the extracted data were imported into STATA version 14 software. First, we extracted the adjusted ORs from all studies that were included. The adjusted odds ratios (AOR) were then pooled using the generic inverse variance method, which involved converting the adjusted odds ratio to a logarithmic scale and then calculating standard error [SE] based on the 95% confidence intervals. The Cochran Q (Standard X^2^) test and Haggin I^2^ statistics were used to assess the presence and degree of heterogeneity among included studies. The tests indicate the presence of average heterogeneity among included studies [I^2^ = 66.7%, *p*-value = 0.006]. Thus, the ORs were pooled using random-effect meta-analysis techniques using the DerSimonian and Laird method, which accounts for the variation between studies [34]. The pooled ORs along with their 95% confidence intervals [CI] were presented using a forest plot.

### Publication bias

In this meta-analysis, possible publication bias was visualized through funnel plots. A symmetrical large inverted funnel resembled the absence of publication biases. Also, the probability of publication biases was tested using Egger's weighted regression test.

### Additional analyses

#### Subgroup analysis and meta-regression

We performed a subgroup analysis based on geographical regions, sampling methods, method of data collection, sample size, publication year, and the type of confounders adjusted in a primary study. Further statistical analyses such as univariate meta-regression were also performed to identify the possible sources of heterogeneity.

#### Sensitivity analysis

Sensitivity analysis using a random-effects model was performed to assess the influence of a single study on the overall pooled odds ratio estimate.

### Operational definition

#### Good menstrual hygiene management

Adolescent girls use a clean menstrual management material to absorb or collect blood that can be changed in privacy as often as necessary for the duration of the menstruation period, using soap and water for washing the body as required, and having access to facilities to dispose of used menstrual management materials.

#### Earning pocket money

In this article, the term "pocket money" refers to any amount of money received by adolescent girls from family or relatives to purchase menstrual hygiene products.

## Results

### Study selection

**Fig. 1:**
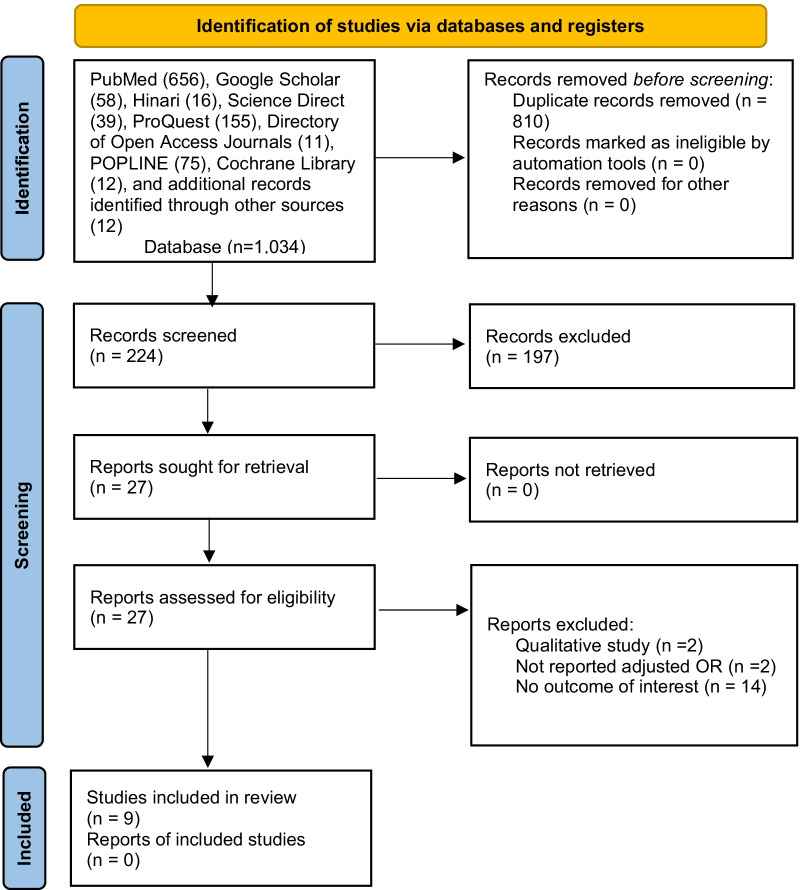
Flow chart of study selection for systematic review and meta-analysis of menstrual hygiene management and its association with earning pocket money among adolescent girls in Ethiopia, 2014–2021

Through all database searches, a total of 1034 studies were found. A total of 810 duplicate records were removed. One hundred ninety-seven articles were excluded after screening 224 studies based on their title and abstract. Then, based on the eligibility criteria, 27 studies were assessed for eligibility. Finally, this systematic review and meta-analysis included 9 studies [10, 23, 25–31] (Fig. 1).

### Study characteristics

As seen in Table 1, the maximum and minimum sample sizes were found in studies conducted by Felleke A., et al. (n = 301) in Eastern Ethiopia [28] and Upashe SP., et al. (n = 828) in Western Ethiopia [25], respectively. This systematic review and meta-analysis comprised a total of 4783 participants. The confounder adjusted odds ratio for examining the link between earning pocket money and menstrual hygiene management was reported in nine of the studies [10, 23, 25–31] (Table 2).

**Table 1:** List of included studies in the systematic review and meta-analysis of the association between earning pocket money and good menstrual hygiene management among adolescent girls in Ethiopia [2014–2021]

Author’s name, year of publication	Study area	Region	Study design	Methods of data collection	Sampling	Included sample size	Response rate	Good MHM	Risk of bias
Kitesa B et al., 2016 [10]	Shoa zone	Oromia	Cross-sectional	Interviewer administered questionnaire	Multistage sampling	430	100	70.2	Low
Habtegiorgis Y et al., 2021[23]	Dessie	Amhara	Cross-sectional	Interviewer administered questionnaire	Multistage sampling	536	98.2	53.9	Moderate
Upashe SP et al., 2015 [25]	Nekemte	Oromia	Cross-sectional	Self-administered structured questionnaire	Simple random sampling	828	98	39.9	Low
Abita Z et al.,2021 [26]	Finot selam	Amhara	Cross-sectional	Self-administered structured questionnaire	A stratified sampling technique	442	99.1	68.8	Low
Anchebi HT et al., 2017 [27]	Adama	Oromia	Cross-sectional	Self-administered structured questionnaire	Multistage stratified sampling	398	94.3	57	Low
Felleke AA et al., 2021 [28]	Harari	Harari	Cross-sectional	Self-administered structured questionnaire	Systematic sampling	301	100	55.8	Moderate
Hasan JH, 2021 [29]	Chelenko	Oromia	Cross-sectional	Self-administered structured questionnaire	Simple random sampling	482	99	68	Moderate
Kedir T, 2017 [30]	West Shoa	Oromia	Cross-sectional	Interviewer administered questionnaire	Multistage stratified sampling	610	98.7	45.6	Low
Biruk E et al., 2018 [31]	Addis Ababa	Addis Ababa	Cross-sectional	Self-administered structured questionnaire	Multistage sampling	756	98	52.5	Low

*SNNPR* South Nations and Nationalities People of the Region; *MHM* Menstrual hygiene management

**Table 2:** Primary studies with available odds ratios of the association between earning pocket money and good menstrual hygiene management among adolescent girls in Ethiopia [2014–2021]

Author’s name, year of publication	Study design	COR [95%CI]	AOR [95%CI]	Reference category	Interpretations of a significant finding	Adjusted confounders
Kitesa B et al., 2016 [10]	Cross-sectional	1.9[1.15–3.12]	1.5[0.87–2.59]	A	Not significant	Educational status, religion, father's educational status, mother's educational status, and access to washing facilities
Habtegiorgis Y et al., 2021 [23]	Cross-sectional	2.66[1.58–4.50]	2.08 [1.15,3.78]	A	Girls who asked for money to purchase pads were two times more likely to practice good menstrual hygiene than those who did not ask [AOR = 2.08, 95% CI: [1.15–3.78]	Age, grade, marital status, live with, maternal education, paternal education, maternal occupation, paternal occupation, regular menses, duration of menses flow, knowledge status, discuss menstrual hygiene with friends, communicate about menstruation with family, and water source functionality in the school
Upashe SP et al., 2015 [25]	Cross-sectional	2.65 [1.76–4.00]	2.73 [1.76,4.26]	A	Girls who earn permanent pocket money from families were nearly three times more likely to have good practice about menstrual hygiene compared to those who don’t earn permanent pocket money from families [AOR = 2.73, 95% CI: 1.76 – 4.26]	Educational status of the mothers, educational status of the father, occupational status of the mother, and monthly income
Abita Z et al.,2021 [26]	Cross-sectional	2.03 [1.35, 3.05]	0.76[0.45,1.29]	A	Not significant	Residency, mother's educational status, father's educational status, father's occupation, wealth index, hearing about menstruation before menarche, discussion with parents about menstruation, learning about menstrual hygiene in the school, and know sanitary pads in the market
Anchebi HT et al., 201s7 [27]	Cross-sectional	1.71[0.84,3.50]	2.27[1.07,4.77]	A	Students whose source of money was their parents were 2.27 times more likely to have a good menstrual hygiene practices than students who earn money by themselves [AOR = 2.27;95% CI = 1.08, 4.77]	Age, mother's educational level, father's educational level, and school uncomfortable to keep hygiene
Felleke AA et al., 2021 [28]	Cross-sectional	0.39[0.24,0.64] *	0.36[0.19,0.65]	B	Adolescent girls who have no permanent pocket money from family [AOR: 0.36:95% CI, 0.31, 0.99] were 64% less likely to have good menstrual hygiene practices than students who have permanent pocket money from family	Grade level, types of school, place of residence, age at menarche, family monthly income, father's educational status, mother's educational status, heard about menstruation before menarche, know sanitary pad, and knowledge about menstrual hygiene practice
Hasan JH, 2021 [29]	Cross-sectional	1.96[1.32,2.90]	2.08[1.27, 3.40]	A	Schoolgirls who earned permanent pocket money from parents or relatives were two times 2.08 times higher odds of practicing good menstrual hygiene than those who have not earned permanent pocket money from parents or relatives [AOR: 2.08, 95%CI: 1.27, 3.40]	Grade level, religion, mother's educational status, knowledge level, residence, ethnicity, age at menarche, and fathers' education
Kedir T, 2017 [30]	Cross-sectional	0.52[0.38, 0.72]	0.62[0.39, 0.97]	B	Adolescent girls who did not receive pocket money were 38% less likely to behave in good menstrual hygiene practice [AOR = 0.62, 95%CI:0.39, 0.97] than adolescent girls who receive permanent pocket money	Age of respondents, residence, place for drying menstrual absorbent material in the household, going to school during menstruation, and taking advice on menstruation
Biruk E et al., 2018 [31]	Cross-sectional	2.17[1.57–3.01]	1.11[0.67,1.81]	A	Not significant	School type, age of the respondents, wealth index, grade, religion, living with, Mothers’ educational status, fathers’ educational status, fathers’ occupation, Mothers’ occupation, and age at first menarche

NA Not Applicable; Reference category “A = NO” indicates: girls who did not receive permanent pocket money from their parents and “B = YES” indicates those girls who receive pocket money; *Crude Odds Ratio [COR] was calculated from a two-by-two table considering girls who did not receive permanent pocket money from their parents as a reference category to estimate the pooled COR [as these studies used different reference category from the rest of the included studies]. Accordingly, the COR [95%CI] for the authors Felleke AA et al., 2021 and Kedir T, 2017 were [COR: 2.54, 95%CI: 1.56,4.11] and [COR:1.91, 95%CI: 1.38, 2.64], respectively

### Risk of bias within studies

When it came to the risk of bias in the studies that were included, the majority of them [99 percent] had a low risk of bias (Additional file 3).

### Meta-analysis

#### Association between earning pocket money and good menstrual hygiene management

**Fig. 2:**
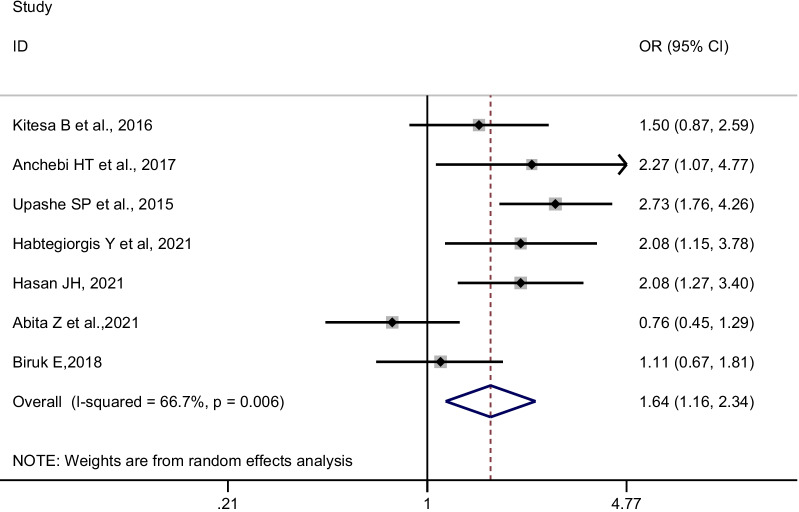
Forest plot of the individual and pooled odds ratios (POR) of the association between earning pocket money and good menstrual hygiene management, 2014–2021

**Fig. 3:**
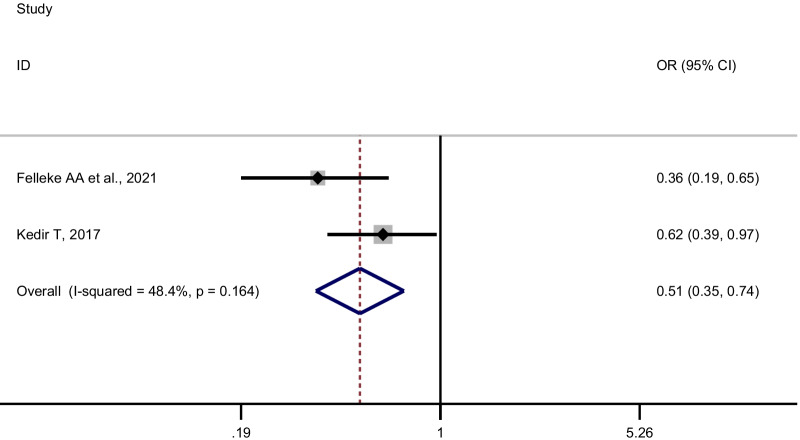
Forest plot of two studies and pooled odds ratios (POR) of the association between earning pocket money and good menstrual hygiene management, 2014–2021

Adolescent girls who received pocket money from their parents or relatives had significantly higher odds of having good menstrual hygiene management than their counterparts (pooled OR [POR] = 1.64, 95%CI: 1.16–2.34, n = 7). We used a random-effects meta-analysis model to estimate POR because the included studies had moderate heterogeneity (I^2^ = 66.7%, *p*-value = 0.006) (Fig. 2). Since two studies [28, 30] used different reference categories, we pooled the confounder adjusted odds ratios separately (Table 2). Likewise, the odds of having good menstrual hygiene management were lower among adolescent girls who did not receive pocket money compared to their counterparts (POR = 0.51, 95% CI: 0.35–0.74; I^2^:48.4%, n = 2) (Fig. 3).

### Additional analysis

#### Subgroup analysis

**Fig. 4:**
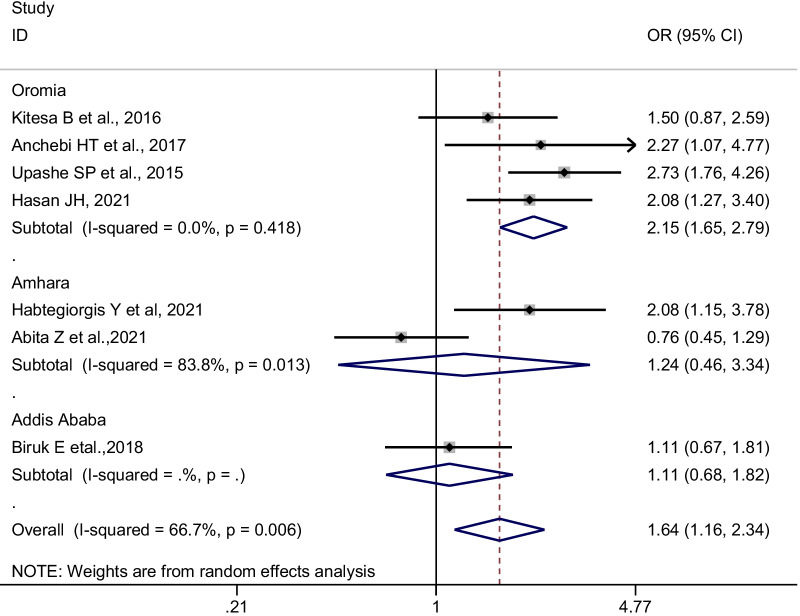
Subgroup pooled odds ratios (POR) of the association between earning pocket money and good menstrual hygiene management among adolescent girls in Ethiopia, by region, 2014–2021

**Fig. 5:**
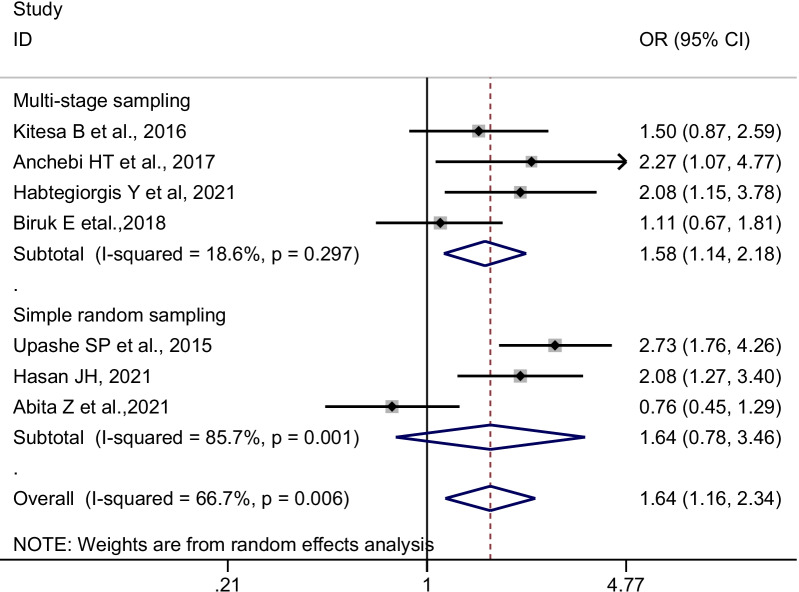
Subgroup pooled odds ratios (POR) of the association between earning pocket money and good menstrual hygiene management among adolescent girls in Ethiopia, by sampling technique, 2014–2021

Subgroup analysis was conducted by geographical regions, sampling methods, method of data collection, sample size, publication year, and confounders adjusted in a multivariate model in primary studies. Accordingly, in the random-effects model, studies conducted in the Oromia region, studies used multistage sampling, and the interviewer-administered data collection technique was found to reveal a significant pooled odds ratio (Figs. 4 and 5). The present meta-analysis also revealed different effect sizes with different sample sizes, and the higher the sample size the more precise the effect size (Table 3).

**Table 3:** Subgroup analysis of the association between earning pocket money is significantly associated with good menstrual hygiene management

Variables	Sub-group	Number of studies	Pooled OR (95%CI)	Heterogeneity tests across the studies	
				I^2^ (%)	*p*-value
Region	Oromia	4	2.14 [1.64–2.79]	0.00	0.418
	Amhara	2	1.24 [0.46–3.34]	83.8	0.013
	Addis Ababa	1	1.11 [0.67–1.81]	-	-
Sampling methods	Multistage sampling	4	1.58 [1.14–2.18]	18.6	0.297
	Simple random sampling	3	1.64 [0.78–3.46]	85.7	0.001
Method of data collection	Self-administered	5	1.60 [0.98–2.62]	76.9	0.002
	Interviewer administered	2	1.74[1.16–2.60]	0.00	0.427
Sample size	≤ 500	4	1.49 [0.91–2.45]	67.5	0.026
	> 500	3	1.85 [1.06–3.23]	72.1	0.028
Publication year	2015–2020	4	1.78 [1.15–2.76]	61.8	0.049
	> 2020	3	1.48 [0.76–2.89]	78.4	0.010
*Confounders adjusted in a multivariable model*					
Knowledge towards menstruation	Adjusted	2	2.08 [1.42–3.04]	0.00	1.00
	Not adjusted	5	1.50 [0.92–2.42]	75.1	0.003
Age	Adjusted	3	1.65 [1.03–2.65]	45.4	0.160
	Not adjusted	4	1.61 [0.94–2.78]	78.9	0.003
Age at menarche	Adjusted	2	1.52 [0.82–2.81]	67.7	0.078
	Not adjusted	5	1.70 [1.06–2.74]	72.6	0.006
Grade level	Adjusted	2	2.08[1.42–3.04]	0.00	1.00
	Not adjusted	5	1.50 [0.92–2.42]	75.1	0.003

#### Meta-regression

A univariate meta-regression model with publication year and sample size as covariates was run to identify potential sources of heterogeneity across primary studies, but none of these variables were found to be statistically significant sources of heterogeneity (Table 4).

**Table 4:** Meta-regression of factors associated with the heterogeneity of the studies included in estimating the pooled effect of earning pocket money on good menstrual hygiene management [based on univariate meta-regression]

Variables	Coef	*p*-value
Sample size	0.0006037	0.627
Year of publication	− 0.0666867	0.408

#### Publication bias

**Fig. 6:**
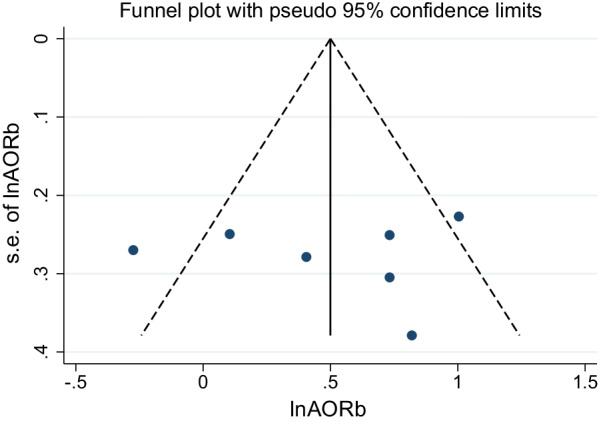
Funnel plot for the meta-analysis of the association between earning pocket money and good menstrual hygiene management, 2014–2021

Using funnel plots, potential publication bias was visualized. The publication bias was also assessed using Egger's tests. The absence of publication biases was represented by an asymmetrical large inverted funnel (Fig. 6). The statistics from Egger's test indicated that there was no statistically significant publication bias, with *p*-values of 0.813.

### Sensitivity analyses

In this review, the sensitivity of each study was checked to identify smaller or larger pooled estimates of the association between earning pocket money and good menstrual hygiene management, which could affect the pooled result by giving wider confidence intervals and variance instability. Based on the result of the random effect model, the sensitivity analyses suggested that the pooled odds ratio or the heterogeneity were somewhat modified after removing a study by Abita Z et al.,2021 [26] (Table 5).

**Table 5:** Sensitivity analysis for the pooled estimates of the association between earning pocket money and good menstrual hygiene management

Author’s name, year of publication	POR	95%CI	I^2^ [%]	*p*-value
Kitesa B et al., 2016 [10]	1.67	1.10–2.53	72.0	0.003
Habtegiorgis Y et al., 2021 [23]	1.58	1.06–2.37	71.2	0.004
Upashe SP et al., 2015 [25]	1.48	1.05–2.10	57.5	0.038
Abita Z et al.,2021 [26]	1.88	1.41–2.49	39.3	0.144
Anchebi HT et al., 2017 [27]	1.58	1.07–2.33	71.0	0.004
Hasan JH, 2021 [29]	1.58	1.04–2.38	70.6	0.005
Biruk E et al., 2018 [31]	1.76	1.20–2.60	66.8	0.010

## Discussion

Menstrual hygiene practice is essential to adolescent schoolgirl empowerment. Improper menstrual hygiene puts girls at risk of infection and impacts students' academic confidence and self-esteem [6, 7, 15]. Menstrual hygiene management is still poorly discussed in Ethiopia, as it is in many developing countries. There has been an increase in evidence on the association between earning pocket money and good menstrual hygiene management [10, 23, 25, 26]. For the first time in Ethiopia, this study tries to assess the association. This meta-analysis found a significant positive relationship between receiving pocket money from parents or relatives and maintaining good menstrual hygiene among adolescent girls.

In this review, adolescent girls who earned pocket money from their families were nearly two times more likely to have good menstrual hygiene than those who don't earn permanent pocket money from their families. This study supported prior studies that show a link between good menstrual hygiene and obtaining pocket money from family members [10, 27, 35]. This might be because girls who earn money from their parents can easily purchase sanitary products for menstrual hygiene, which may lead girls to have good menstrual hygiene [16]. According to prior studies, girls from families with higher monthly household expenditures or belonging to the richest wealth quintile were also more likely to use sanitary napkins and have good menstrual hygiene management than their counterparts [7, 31, 36].

This study found pocket money to be a crucial component in addressing hygienic management of menstruation among schoolgirls. In this regard, recent literature has recognized pocket money as an important intervention to address girls' menstrual hygiene needs [37], as well as access to quality hygiene supplies to promote good menstrual hygiene [38].

Studies suggest that menstruation contributes to school absenteeism, possibly extending to school dropout. Lack of menstrual hygiene resources was reported to lead directly to drop out or school absence [18, 37, 39]. According to a review conducted in India, females miss school due to the fear and embarrassment of blood and body odor leaking [16]. In fact, poverty was a constant underlying attribute in the stories of the girls either directly or indirectly responsible for the higher proportion of absenteeism or dropout. A review discovered that limited financial resources to purchase supplies continue to be a barrier to improving menstrual hygiene management in many resource-limited settings [40]. As a result, poor menstrual hygiene remains a major impediment to girls' education and their self-esteem and personal development [41].

Our review demonstrated that adolescent girls who did not get pocket money were less likely than their peers to adopt proper menstrual hygiene management. According to studies conducted in Kenya [37] and Tanzania [42], girls frequently mention the need for sanitary pads, but their parents' funds rarely allow them to buy the high-quality or sufficient number of pads required, and girls' financial dependence on sanitary materials impacts their MHM, school attendance, and sexual behaviors. We believe that earning permanent pocket money from parents may, in turn, enhance girls' menstrual hygiene, which may lead to increased school attendance. Eliminating menstrual needs as a burden for schoolgirls will improve their dignity, school involvement, and possibly their menstrual hygiene management. Moreover, access to hygienic menstrual management methods for adolescent girls is critical from both public health and adolescent girls' perspectives. One strategy for this is to give schoolgirls pocket money to spend on their menstrual hygiene needs as needed.

## Limitations

Our study has several limitations. First, all included studies were cross-sectional studies, so causality cannot be inferred. Second, our study could lack representativeness at a country level as we did not find a study from some regions of the country. Third, the included studies were not free of social desirability bias. Fourth, there was average heterogeneity between included studies as indicated by the I^2^ statistic. This might be explained by the methodological variation and/or difference in the study setting. Hence, we performed sub-group analysis, sensitivity analysis, and meta-regression to explore the variations within the studies. Finally, we did not examine the link between the amount of pocket money and the menstrual hygiene of girls. However, we believe that the amount of pocket money a girl has may affect her menstrual hygiene, and further research is necessary to address this topic in depth.

## Conclusions

The evidence suggests that adolescent girls who earned pocket money were more likely to practice good menstrual hygiene management. Progress toward improving schoolgirl menstrual hygiene will necessitate consideration of this factor to enhance menstrual hygiene practice.

## Supplementary Information

**Additional file 1:** PRISMA checklist.

**Additional file 2:** Examples of searching strategy.

**Additional file 3:** Risk of bias for included studies.

**Additional file 4:** STATA data file.

## Data Availability

All data generated or analyzed during this study are included in this published article and its Additional files (Additional file 4).
