# Supplementary material for: Earning pocket money and girls’ menstrual hygiene management in Ethiopia: a systematic review and meta-analysis
Source: BMC Womens Health. 2022 Jul 4;22:271. doi: 10.1186/s12905-022-01855-2 (PMC9254547; doi:10.1186/s12905-022-01855-2)
Supplement: Supplementary file 2 — Additional file 2: Examples of searching strategy. [file 12905_2022_1855_MOESM2_ESM.docx]

**Supplementary file 2: Examples of searching strategy**

| **Database** | **Example of searching strategy** |
| --- | --- |
| PubMed | ((((((((((Adolescent [MeSH Terms]) OR Adolescents [Text Word]) OR adolescent) OR adolescence) OR puberty) OR peer) OR school)) AND (((Menstruation [MesH]) OR menstrual) OR menses)) AND (((((((Hygiene [MeSH]) OR hygiene) OR hygienically) OR sanitation) OR sanitary) OR Feminine Hygiene Products [MesH]) OR Menstrual Hygiene Products [MesH])) AND ((Ethiopia [MeSH Terms]) OR Ethiopia) |
| POPLINE | (Menstrual OR Menstruation OR "menstrual hygiene") AND "Ethiopia" |
| Google Scholar | (“menstrual” OR “hygiene”) AND "Ethiopia" |
| Science Direct | “Menstrual hygiene" AND "Ethiopia” |
| Hinari | (“Menstrual hygiene management” OR “Menstrual hygiene” OR “Menstrual hygiene practice” OR "Menstrual" OR "Hygiene") AND Ethiopia |
| ProQuest | “Menstrual hygiene" AND "Ethiopia” |
| African Journal Online | “Menstrual hygiene" AND "Ethiopia” |
| Direct of Open Access Journals | “Menstrual hygiene" AND "Ethiopia” |
| Cochrane Library | ("Menstrual blood loss [MeSH]" OR "mensural) AND Ethiopia |
