# Supplementary material for: Earning pocket money and girls’ menstrual hygiene management in Ethiopia: a systematic review and meta-analysis
Source: BMC Womens Health. 2022 Jul 4;22:271. doi: 10.1186/s12905-022-01855-2 (PMC9254547; doi:10.1186/s12905-022-01855-2)
Supplement: Supplementary file 3 — Additional file 3: Risk of bias for included studies. [file 12905_2022_1855_MOESM3_ESM.docx]

**Supplementary File 3:** Risk of bias for included studies

| **Author’s name, year of publication** | **Sampling frame** | **Sampling strategy** | **Sample size** | **Description of research setting & population** | **Data analysis conducted with sufficient coverage** | **Valid methods used for the identification of the condition** | **Reliability of the instrument used** | **Statistical analysis methods** | **Response rate** | **Total** | **Risk of bias** |
| --- | --- | --- | --- | --- | --- | --- | --- | --- | --- | --- | --- |
| Felleke AA et al., 2021 | 1 | 0 | 0 | 0 | 0 | 1 | 1 | 1 | 0 | 4 | Moderate |
| Kitesa B et al., 2016 | 0 | 0 | 0 | 0 | 0 | 1 | 1 | 0 | 0 | 2 | Low |
| Anchebi HT et al., 2017 | 0 | 0 | 0 | 0 | 0 | 1 | 1 | 0 | 0 | 2 | Low |
| Upashe SP et al., 2015 | 0 | 0 | 0 | 0 | 0 | 1 | 1 | 0 | 0 | 2 | Low |
| Habtegiorgis Y et al, 2021 | 0 | 0 | 0 | 0 | 0 | 1 | 1 | 0 | 0 | 2 | Low |
| Hasan JH, 2021 | 0 | 0 | 0 | 0 | 0 | 1 | 1 | 0 | 0 | 2 | Low |
| Abita Z et al.,2021 | 0 | 0 | 0 | 0 | 0 | 1 | 1 | 0 | 0 | 2 | Low |
| Kedir T, 2017 | 0 | 0 | 0 | 0 | 0 | 1 | 1 | 0 | 0 | 2 | Low |
| Biruk E et al., 2018 | 0 | 0 | 0 | 0 | 0 | 1 | 1 | 0 | 0 | 2 | Low |
